# Characterization of Nonjunctional Hemichannels in Caterpillar Cells

**DOI:** 10.1673/031.011.0106

**Published:** 2011-01-18

**Authors:** Kaijun Luo, Matthew W. Turnbull

**Affiliations:** Department of Entomology, Soils, and Plant Sciences, Clemson University, Clemson, South Carolina 29634-03 15, USA; ^1^ Current Address: School of Life Sciences, Yunnan University, Kunming, Yunnan 650091, China

**Keywords:** gap junction, hemichannel, hemocyte, innexin

## Abstract

Recent studies have demonstrated that hemichannels, which form gap junctions when paired from apposing cells, may serve additional roles when unpaired including cell adhesion and paracrine communication. Hemichannels in mammals are formed by connexins or pannexins, while in insects they are formed by pannexin homologues termed innexins. The formation of functional gap junctions by insect innexins has been established, although their ability to form functional nonjunctional hemichannels has not been reported. Here the characteristics of nonjunctional hemichannels were examined in three lepidopteran cell types, two cell lines (High Five and Sf9) and explanted hemocytes from *Heliothis virescens* (Fabricius) (Lepidoptera: Noctuidae). Selective fluorescent dye uptake by hemichannels was observed in a significant minority of cells, using fluorescence microscopy and flow cytometry. Carbenoxelone, an inhibitor of mammalian junctions, disrupted dye uptake, while flufenamic acid and mefloquine did not. The presence of Ca^2+^ and Mg^2+^ in the media increased hemichannel activity. Additionally, lipopolysaccharide, a stimulator of immune activity in lepidopterans, decreased dye uptake. These results demonstrate for the first time the activity of nonjunctional hemichannels in insect cells, as well as pharmacological tools to manipulate them. These results will facilitate the further examination of the role of innexins and nonjunctional hemichannels in insect cell biology, including paracrine signaling, and comparative studies of mammalian pannexins and insect innexins.

## Introduction

Gap junctions provide direct transfer of small molecules between adjacent cells in multicellular animals. Although functionally conserved across broad phylogeny, gap junctions are encoded by two multigene families: connexins are restricted to chordates, while the pannexin gene family encodes junctional proteins in both vertebrates and invertebrates. Pannexins, which were recently identified in mammalian genomes (Panchin et al. 2000), are referred to in insects and other invertebrates as innexins (invertebrate con_nexins_). Connexin-based gap junctions consist of a pair of connexons or hemichannels, each provided by a single apposing cell of the pair, and each of which is comprised by six monomeric connexins (Yeager and Harris. 2007; Unger et al. 1999). Mammalian genomes encode approximately 20 connexins and 3 pannexins genes, which exhibit gene- and tissue-specific expression patterns (Willecke et al. 2002; Panchin et al. 2000). Insect genomes encode multiple innexin loci as well. For example, the *Drosophila melanogaster* genome encodes eight *innexin* loci (Stebbings et al. 2002), which display distinct expression patterns (Phelan et al. 1998a, 1998b; Stebbings et al. 2002; Lehmann et al. 2006) and a lack of functional redundancy (Curtin et al. 2002).

Gap junctions aid in the coordination of multicellular activities through the selective transfer of cytoplasmic metabolites, including nucleotides, ions such as Ca^2+^, lipid derivatives, and small peptides (Harris 2001). Connexin gap junction intercellular communication is involved in many physiological processes including left-right axis patterning (Levin 2007; Levin and Mercola. 1998), coordination of cell replication and death (Doble and Kardami 1995; Kalvelyte et al. 2003), antigen presentation in immunocytes (Neijssen et al. 2005), and neuronal adhesion and migration (Elias et al. 2007). Considering the likely evolutionary homology of pannexins and innexins (Baranova et al. 2004; Yen and Saier. 2007), they may share physiological roles or at least provide insight into one another. The roles of pannexin-mediated junctional communication are less clear than those that are connexin-mediated, but may include neural synchronization (Barbe et al. 2006). Gap junction-mediated communication is important in a wide range of insect physiological processes including epithelial morphogenesis (Bauer et al. 2004) and organogenesis (Bauer et al. 2002; Bauer et al. 2001), oocyte survival and maturation (Gilboa et al. 2003; Waksmonski and Woodruff. 2002), and activity of electrical synapses (Phelan et al. 1996) and Malpighian tubules (Weng et al. 2008).

The occurrence and function of nonjunctional (unapposed) hemichannels are less clear than those of gap junctions. Nonjunctional connexin hemichannels may coordinate cellular behaviors via paracrine signaling (e.g., by ATP and glutamine release) (Stout et al. 2002; Zhao et al. 2005), as well as affecting aggregation and adhesion (Cotrina et al. 2008). The activity levels of nonjunctional connexin hemichannels can change dynamically based on cellular conditions and the environment including pH and presence and concentration of certain ions (Contreras et al. 2001; De Vuyst et al. 2006, 2007; Retamal et al. 2007; Zhang et al. 2006). In contrast to connexins, the primary role of pannexins may be to generate nonjunctional hemichannels (Dahl and Locovei. 2006), and innexins have been demonstrated to form functional nonjunctional channels in leech neuronal tissue (Bao et al. 2007). It therefore seems likely that nonjunctional hemichannels may provide paracrine-mediated coordination of cellular activities in invertebrates, analogous to the hemichannels of mammals.

Gap junctions and/or innexin expression occurs in a wide array of insect tissues. Given the breadth of expression, the activity of nonjunctional hemichannels in leech tissue, and the functional similarities between innexins, pannexins, and connexins, it is highly likely that nonjunctional hemichannels occur in insects and may represent an as yet unreported communication modality. To address this possibility, we examined two lepidopteran cell lines and hemocytes for nonjunctional hemichannel activity using dye uptake assays. We observed activity of nonjunctional hemichannels in all three cell types and have noted the efficacy of several common pharmacological inhibitors on these hemichannels, which will be useful for future functional analyses. This work will facilitate future studies to examine the roles of insect hemichannels in insect biology, and comparisons to the chordate pannexins.

## Materials and Methods

### Cell culture and hemocyte isolation

High Five (BTI-TN-5B1-4), from *Trichoplusia ni* (Hübner) (Lepidoptera: Noctuidae) and Sf9 cells (from *Spodoptera frugiperda* (J.E. Smith) (Lepidoptera: Noctuidae) were obtained from Invitrogen Corp. (http://www.invitrogen.com) and maintained as adherent cells in TnMFH media (Mediatech, www.cellgro.com) supplemented with 5% FBS. Cells were grown to confluency, harvested and washed 3 × 5 min in either TnMFH serum free media (SFM), or in Hanks' buffered salt solution (HBSS: 0.4 g/L KCl, 0.06 g/L KH_2_PO4, 8 g/L NaCl, 0.0477 g/L Na_2_HPO4, 0.35 g/L NaHCO3, 1 g/L glucose, pH 7.1) less Mg^2+^ and Ca^2+^ (divalent cation free, DCF), or HBSS plus 0.8 mM Mg^2+^ and 1.3 mM Ca^2+^ (divalent cation containing, DCC). Cell number and viability counts were performed prior to all assays using standard trypan blue staining and hemocytometer protocols. *Heliothis virescens* (Fabricius) (Lepidoptera: Noctuidae) larvae were obtained from Dr. Linda Gahan (Clemson University) and maintained as previously detailed (Gould et al. 1995), or were obtained commercially as second instar larvae (Bio-Serv, www.bio-serv.com). Fourth instar larvae were bled from an incision of abdominal proleg into serum-free TnMFH containing 200 µg/ml reduced glutathione to block melanization. Hemolymph was pooled from twenty larvae, washed with TnMFH SFM, and cell number and viability determined.

### Dye uptake assays at 4°C

Dye uptake assays were performed to visualize the activity of nonjunctional hemichannels, using a variety of fluorescent dyes with different physical and chemical characteristics (Table 1). Stock solutions of ethidium bromide (EB, 0.5 mg/ml; Sigma, www.sigmaaldrich.com), 4′-6-diamidino-2-phenylindole (DAPI, 30 mM; Sigma), propidium iodide (PI, 1 mg/ml; Sigma), and lucifer yellow (LY, dilithium salt, 4 mg/ml; Sigma) were prepared in sterile ddH_2_O. Cells prepared as above were seeded in 96-well plates at 10^4^ cells/well and incubated at 4 °C for 2 h, then 300 nM of DAPI, 5 µg/ml of EB, 50 µg/ml of PI, or 200 µg/ml LY fluorescence dye was added, dependent on assay design. After incubation at 4 °C for 5 min (cells were incubated in LY for 4 hrs, as discussed below), cells were washed 5 times with cold PBS, and fixed for 15 min in 3.7% formaldehyde. Cells were analyzed using fluorescence microscopy (below).

### Dye uptake assays at room temperature (23°C)

**Table 1.**
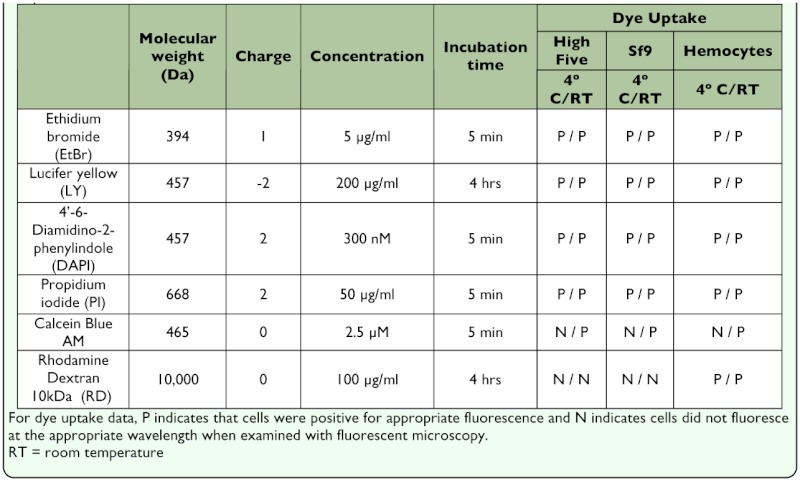
Characteristics and uptake of six fluorescent dyes in three different insect cell types at 4°C and room temperature.

Rhodamine dextran (RX), Sigma) was prepared as a 10 mg/ml stock solution in sterile ddH2O. Calcein Blue AM (Invitrogen) was prepared as a 5 mM stock solution in DMSO. High Five, Sf9, and hemocytes prepared as above were transferred to a 96well tissue culture plate at 10^4^ cells per well in appropriate media (TnMFH SFM, HBSS DCF, HBSS DCC). For analysis by flow cytometry, LY and RD were added to 60 µg/ml and 100 µg/ml, respectively. The plate was incubated with gentle rocking at room temperature for 1 h, and then cells were examined by flow cytometry (below). For fluorescence microscopy, LY was added to 200 µg/ml and the plate incubated with gentle rocking at room temperature for 4 hrs, prior to washing cells with the appropriate media; a longer incubation period and higher concentration were required with LY to conclusively distinguish dye uptake by microscopy. Suspected pharmacological modifiers of hemichannel activity were also tested. In those assays, cells were incubated for 30 min at room temperature in media containing carbenoxelone (CBX, 10 mM stock in sterile ddH_2_O; Sigma), flufenamic acid (FFA, 10 mM stock in DMSO; Sigma), mefloquine (MFO, 10 mM stock in 10% DMSO; Sigma), or lipopolysaccharide (*E. coli* LPS, 1 mg/ml stock in sterile ddH_2_O; Sigma), washed with TnMFH or HBSS, fresh media including dye was added, and cells processed as follows.

### Flow cytometry

Flow cytometry was performed using a Guava EasyCyte Plus System, Millipore (www.millipore.com). Unlabeled High Five or Sf9 cells were used to gate fluorescence for either cell line, respectively. LY is gap junction permeable, while neither LY nor 10 kDa RD are membrane permeant, and RD is not gap junction permeant. Therefore, LY/RD- cells were considered unloaded, LY+/RD- cells were considered to have active hemichannels, LY-/RD+ considered to be endocytically active, and LY+/RD+ considered to be nonviable. The proportion of cells with active hemichannels was calculated as the ratio of (LY+/RD-) cells / total viable cells. Five thousand events were captured per replicated treatment, and each assay was repeated with at least three independent replicates.

### Fluorescence microscopy

For microscopic analysis of dye uptake, cells were stained with calcein blue AM (2.5 µM final concentration) for 5 min at room temperature to verify viability following chemical presentation and dye loading. Samples were examined using a Nikon TE2000 inverted epifluorescence microscope equipped with DAPI, FITC, and TRITC filter sets and a Nikon DS2 monochrome camera. Samples stained with a single fluorophore (calcein blue AM, LY, or RD) were initially visualized to ensure lack of spectral overlap for paired fluorophores. A minimum of 125 cells and three non-overlapping fields of view were imaged per well, and three independent replications were performed for each treatment. Images were analyzed using Nikon Elements 2.0 (www.nikon.com). The proportion of cells with active hemichannels was calculated as the ratio of cells with both LY and calcein blue AM to cells with calcein blue AM only.

### Statistical analyses

Statistical comparisons of experimental means to control means were performed using independent samples 2-tailed *t*-test with SPSS 16.0 for Windows (SPSS Inc.).

## Results

### Lepidopteran cells take up hemichannel permeant dyes

**Figure 1.**
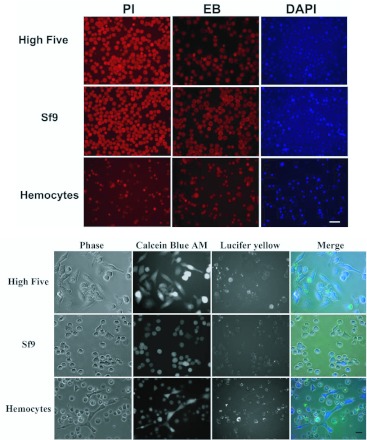
Lepidopteran cells exhibit dye uptake suggestive of nonfunctional hemichannel activity. (A) High Five, Sf9, and primary culture *H. virescens* hemocytes are capable of uptake of PI, EB, and DAPI at 4° C, suggesting that endocytosis is not responsible for dye uptake. Scale bar = 50 µm. (B) Lucifer yellow uptake at room temperature occurs in all three cell types, as visualized by fluorescence microscopy; Calcein Blue AM staining indicates viability of the cells. Scale bar = 20 µm. All assays were performed in TnMFH SFM. High quality figures are available online.

The uptake of dyes which are non-membrane permeable by cells can be indicative of unapposed hemichannel activity, although hemichannels may also be selectively permeable to dyes. Therefore the ability of lucifer yellow (LY), DAPI, propidium iodide (PI), and ethidium bromide (EB), which have very limited membrane permeability, were assayed for uptake. The large, non-membrane permeable molecule rhodamine dextran 10 kDa (RD), was also used as a marker for phagocytic activity, as well as the membrane permeant marker calcein blue AM, as a viability marker. Based on similar studies with mammalian systems (e.g., Page et al., 1994), we predicted that LY uptake and cellular distribution might vary between homogeneous and heterogeneous cytoplasmic localization. On the other hand, hemichannel-mediated uptake of DAPI, PI, and EB was expected to result in homogeneous cytoplasmic distribution of fluorescence (Valiunas, 2002). LY uptake was observed in pilot investigations using fluorescence microscopy, although discrimination from background fluorescence was difficult. To optimize the assay further the effect of interval post-plating was examined on dye uptake in Sf9 and High Five cells. Cells exhibited little to no uptake of dyes (PI, EB, DAPI, and LY) greater than 12 h post-plating (data not shown), suggesting little to no activity of nonjunctional hemichannels. At fewer than 12 h after plating, both cell lines exhibited uptake of all four tested dyes. Therefore, all assays reported here used freshly passaged (<12 h) cells. Furthermore, as LY was difficult to differentiate from background after 1 h incubation, microscopic analysis of that dye (along with simultaneous incubation with RD to identify phagocytotically active cells) was performed after 4 h incubation, while other molecules (PI, EB, and DAPI) were incubated for 5 min (Table 1, Figure 1A).

High Five and Sf9 cells were examined using flow cytometry for their ability to take up the membrane impermeant molecules LY and RD at room temperature, in TnMFH SFM. Sf9 cells exhibited a slightly higher percentage of LY-positive cells under control conditions than High Five cells (27±3.1% and 23±2.5%, respectively; n=3) (Table 2). Few cells were positive for RD in these assays (data not shown), suggesting that dye uptake was hemichannel- and not phagocytosis-mediated. Cells were then analyzed for dye uptake and morphological analysis at room temperature using fluorescence microscopy with LY and calcein blue AM; positive staining with the latter verified cell viability. A large percentage of cells were positive for both LY and calcein blue AM, suggestive of active nonjunctional hemichannels in many of the cells. LY staining frequently was punctate, reminiscent of LY uptake and cellular distribution in rat myocytes (Page et al., 1994). Approximately half of all hemocytes exhibited some level of LY uptake, as well (Table 2, Figure 1B). Although not quantified, it appears that while granulocytes exhibited ready dye uptake, plasmatocytes rarely did (Figure 1B). Fluorescence microscopy consistently resulted in a higher percentage of LY-positive cells than flow cytometry (Table 2), likely due to the increased concentration and incubation period used in the former.

To rule out phagocytosis as a mode of uptake, phagocytosis was inhibited by performing a subset of assays at 4° C. LY, PI, EB, and DAPI all entered Sf9 and High Five cells at rates similar to those observed at room temperature (data not shown). This, combined with the rarity of RD-positive cells in flow cytometry assays, strongly suggests that endocytosis is not responsible for a significant portion of dye uptake in these assays.

### Known pharmacological blockers of hemichannels inhibit dye uptake

**Figure 2.**
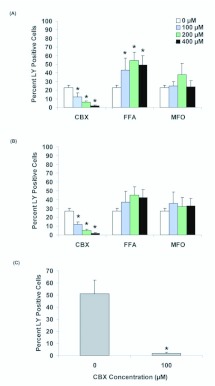
Alteration of dye uptake activity following treatment of cells with the gap junction inhibitors carbenoxelone (CBX), flufenamic acid (FFA), and mefloquine (MFO). (A) High Five and (B) Sf9 cell lines were analyzed by flow cytometry, and exhibit differential response to the three blockers in TnMFH SFM. (C) Mean dye uptake in hemocytes following exposure to carbenoxelone. Data are representative of 3 replicates performed in TnMFH SFM, and variance is s.e.m. Means indicated by asterisk significantly differ from untreated control, *p*< 0.05. High quality figures are available online.

Carbenoxelone, flufenamic acid, and mefloquine are potent gap junction inhibitors in mammalian systems, and carbenoxelone inhibits nonjunctional hemichannel activity in leech neuronal tissue (Bao et al. 2007). The ability of these agents to block dye uptake in insect cells was tested, both to further test the likelihood that hemichannels mediate dye uptake and to identify modifying reagents. Pre-incubation of cells with carbenoxelone significantly reduced the percentage of LY-positive cells in TnMFH for both lines (Figure 2A, B). Flufenamic acid led to a slight (Sf9) to significant (High Five) increase in dye uptake, while mefloquine did not alter hemichannel-mediated uptake of LY. Carbenoxelone consistently inhibited dye uptake in the two cultured lines, and was found to significantly reduce LY uptake in hemocytes at 100 µM (Figure 2C). Viability was indistinguishable between control and chemical treated cells at 100 µM for all three cell types (data not shown). Flufenamic acid and mefloquine were not tested in hemocytes, given their lack of inhibitory activity in cell culture and the sensitivity of hemocytes to carbenoxelone.

**Table 2.**
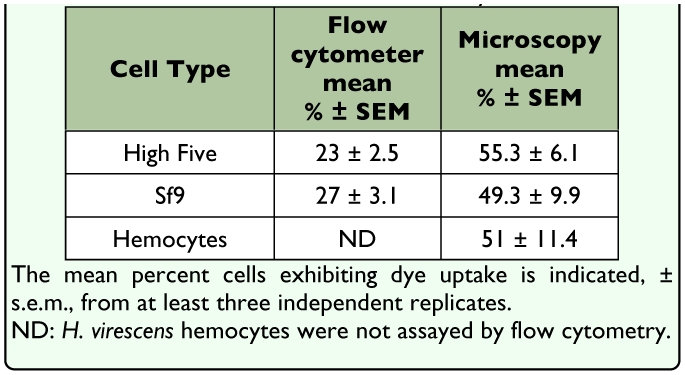
Lucifer yellow uptake observed in High Five and Sf9 cell lines and isolated *Heliothis virescens* hemocytes, in TnMFH.

**Figure 3.**
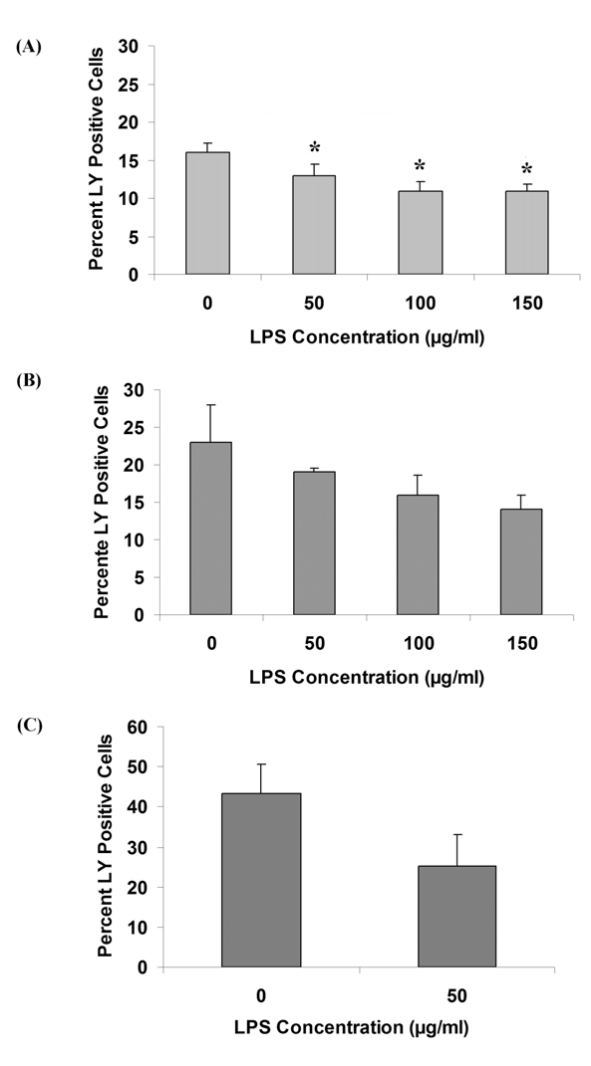
Ca^2+^ and Mg^2+^content of the media significantly affects LY uptake. (A) High Five, (B) Sf9, and (C) hemocytes were examined for LY uptake in 1.3 mM Ca^2+^ and 0.8 mM Mg^2+^ (DCC), and 0 mM Ca^2+^ and Mg^2+^ (DCF) HBSS. Bars represent the mean percent of LY positive cells, scored by (A– B) flow cytometry or (C) fluorescence microscopy. (D) Hemocytes incubated in DCC and DCF media. Scale = 20 µm. Data are presented as means with s.e.m., from three independent replicates. Asterisk indicates that the mean of DCC treated cells significantly differs from DCF treatment at p< 0.05. High quality figures are available online.

### Media Ca^2+^ and Mg^2+^ affect dye uptake

Reduced extracellular Ca^2+^ concentration is correlated with increased hemichannel activity for several connexin-based hemichannels (Li et al. 1996; Verselis and Srinivas. 2008), and alteration of intracellular Ca^2+^ affects leech nonjunctional hemichannel activity (Bao et al. 2007). Hanks' buffered salt solution (HBSS), either with 1.3 mM Ca^2+^ and 0.8 mM Mg^2+^ (DCC) or without the two cations (DCF), was used to examine the effect of the cations on lepidopteran hemichannels. High Five and Sf9 cells were examined for LY uptake using flow cytometry in both DCF and DCC. Incubation of cells in DCC resulted in increased dye uptake for both lines relative to cells incubated in DCF (Figure 3A, B). *H. virescens* hemocytes also exhibited a significant increase in LY uptake in DCC relative to DCF, as demonstrated with fluorescence microscopy (Figure 3C, D), suggesting that cation presence may be a positive regulator of lepidopteran hemichannel activity.

### LPS reduces hemichannel activity

**Figure 4.**
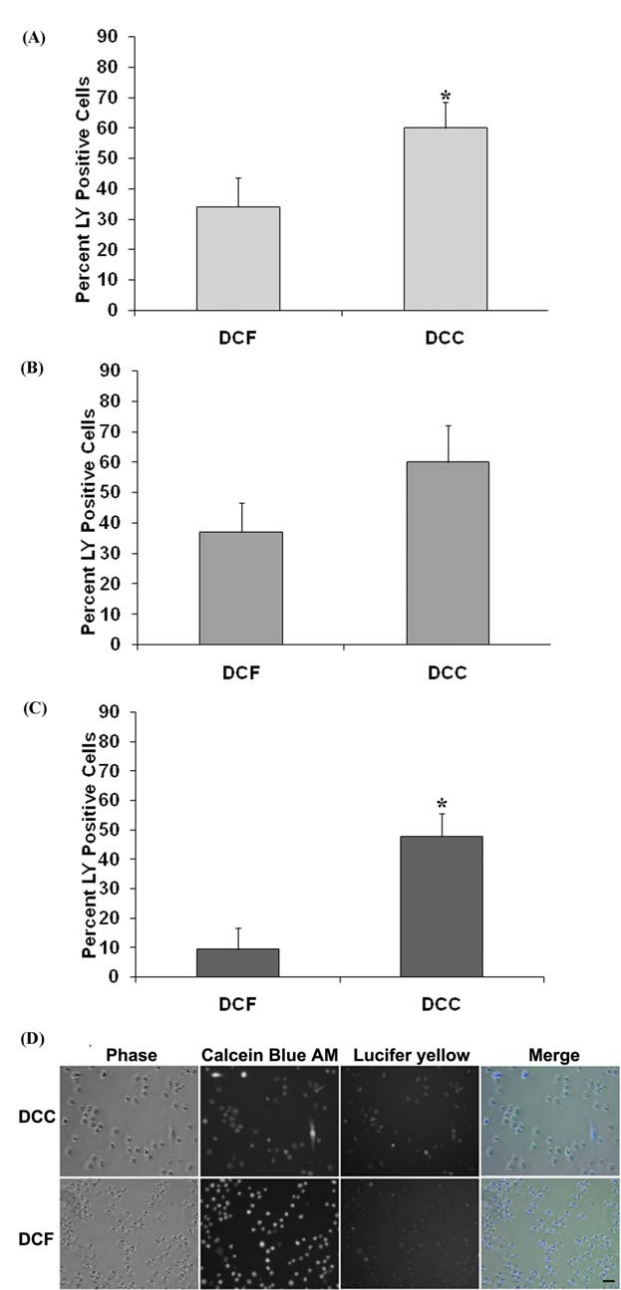
LPS reduces dye uptake in a dose-dependent fashion. (A) High Five cells exhibit a significant decrease in dye uptake in the presence of LPS, while (B) Sf9 cells and (C) *H. virescens* hemocytes exhibit a non-significant reduction. Data were obtained by (A, B) flow cytometry and (C) fluorescence microscopy. Data are presented as means with s.e.m., from three independent replicates. Means indicated by asterisk significantly differ from untreated control, *p*< 0.05. High quality figures are available online.

In mammals, inflammatory stimuli lead in some cases to altered gap junctional and hemichannel activity in immunocytes. As hemocytes are the primary cellular components of the lepidopteran immune response, the effect of *E. coli* lipopolysaccharide (LPS) on hemichannel activity was examined. The presence of LPS reduced LY uptake in a concentration-dependent fashion in all three cell types (Figure 4). Flow cytometry showed that High Five cells were relatively sensitive to the application, exhibiting significant decreases in hemichannel activity at lower concentrations (Figure 4A) than Sf9 (Figure 4B). Fluorescence microscopy showed that preincubation with LPS similarly reduced LY uptake by *H. virescens* hemocytes (Figure 4C). Although dye uptake was reduced in LPS-treated Sf9 and *H. virescens* hemocytes, neither significantly differed from untreated cells.

## Discussion

Although gap junction intercellular communication is important in a wide variety of insect physiological processes (e.g., electrical synapse activity, organogenesis, yolk transfer, gamete viability) (Krishnan et al. 1993; Phelan et al. 1996; Bauer et al. 2001; Tazuke et al. 2002; Waksmonski and Woodruff. 2002), the activity and roles of nonjunctional hemichannels in insects have not been reported. However, leeches, which lack connexins, exhibit nonjunctional hemichannel activity as demonstrated by dye uptake and electrophysiological assays (Bao et al. 2007), suggesting that insects may have active nonjunctional hemichannels as well. Therefore three lepidopteran cell types were tested to examine the presence and activity of insect hemichannels. High Five cells, which are derived from *T. ni*, exhibit several characteristics of lepidopteran immunocytes (granulocytes), and are an *in vitro* model for hemocyte behavior (Beck and Strand. 2003). Sf9 cells are derived from pupal ovaries of *S. frugiperda*, and exhibit many endothelial characteristic; they have previously been used for heterologous expression of connexins (Beahm et al. 2006; Oshima et al. 2003; Stauffer 1995). The hemocytes isolated from *H. virescens* represent a mixed population of plasmatocytes and granulocytes, the primary immunocytes, as well as several minor populations (Lavine and Strand. 2002; Strand 2008; Davies et al. 1987). All three cell types have previously been demonstrated to form gap junctions and/or express innexins (Bukauskas et al. 1997; Epstein and Gilula. 1982; Turnbull et al. 2005). These study cells therefore include a range of cell origin and phenotype, and should provide a basic understanding of the distribution of nonjunctional hemichannels among several physiological systems. The findings, that lepidopteran cells exhibit nonjunctional hemichannel activity and that commonly used pharmacological and biological agents may affect their activity, are important in considering communication modalities in relevant physiological systems.

Partial inactivation of dye uptake by carbenoxelone was observed, but not for flufenamic acid and mefloquine, with effects varying by cell type and chemical concentration (Figure 2). Such variation may be due to the relative sensitivities of the constituent proteins (i.e., the sensitivity may be dependent on the particular constituent innexins), as is seen in the rabbit retina (Pan et al., 2007). Variability also was observed in response to inhibitors between pannexins and connexins: pannexins are relatively insensitive to flufenamic acid, while it is a potent blocker of the connexins (Pelegrin and Surprenant, 2006).

The Ca^2+^ and Mg^2+^ content of the media was found to have a strong effect on LY uptake. Increasing intracellular Ca^2+^ increased leech innexin nonjunctional hemichannel activity (Bao et al. 2007), while the presence of extracellular divalent cations generally is correlated with inhibition of connexin hemichannel activity (Verselis and Srinivas. 2008; Pfahnl and Dahl. 1999). Our results suggest a complex sensitivity that is potentially dependent on the innexin composition of the hemichannel, as has been postulated to be a basis for variation between mammalian gap junction sensitivities (Verselis and Srinivas. 2008). Additionally, our assays were generally performed in TnMFH SFM, which has Ca^2+^ and Mg^2+^ concentrations (9 mM and 2.2 mM, respectively) in the physiologically relevant range for these cell types. Although this Ca^2+^ level is higher than that reported for some lepidopterans, the Mg^2+^ concentration is much lower than that reported (Bindokas and Adams. 1988). The relatively high Mg^2+^ and Ca^2+^ levels in lepidopteran hemolymph, and the response of hemichannels to ion level, suggests there may be unique regulators of hemichannel activity relative to ion levels for lepidopterans. This possibility remains to be tested. But, regardless, the tested ion concentrations (in TnMFH and HBSS), coupled with our assay method (i.e., an endpoint analysis of dye uptake, rather than rate or concentration analysis), may explain the relatively high basal hemichannel activity found.

Until recently it was thought that nonjunctional hemichannels typically must reside in closed state, to avoid loss of cell homeostasis (Bennett et al. 2003). We utilized longer duration studies and found that a significant portion of the population of all three cells have at least intermittently open hemichannels. Although incapable of discriminating the duration of open state or the percent of open hemichannels, our methods verified that pharmacological and endotoxin modifiers are capable of sustained inhibition. Rate comparison studies are currently underway to refine this view of opened versus closed states.

Previous studies in mammalian (as reviewed in (Neijssen et al. 2007; Saez et al. 2000)) and insect systems (Churchill et al. 1993; Grimstone et al. 1967; Turnbull et al. 2005) have identified gap junctions or their encoding proteins in immunocyte populations. Interestingly, we found that all three investigated cell types exhibited a reduction in hemichannel activity following LPS application, a common model for immune stimulation. In mammalian leukocytes, LPS application may lead to connexin expression levels being down-regulated (De Maio et al. 2000; Temme et al. 2000; Fernandez-Cobo et al. 1999), up-regulated (Temme et al. 2000), unaffected (Oviedo-Orta et al. 2000), or post-translationally modified (De Maio et al. 2000; Gingalewski et al. 1996). Multiple innexins are expressed by the cells examined here (Turnbull et al., unpublished data), and it seems probable, by analogy to connexins and pannexins, that different innexins are differentially affected by inflammatory state. Leech hemichannels release ATP to activate and recruit microglia, immune cells of the central nervous system, to the site of injury (Samuels et al., 2010). Many signal transduction pathways involved in immune responses are broadly conserved phylogenetically (Gupta, 1991a, 1991b; Zakarian et al. 2003; Schmidt et al. 2008; Strand 2008), and we propose that hemichannels may play a similar role in regulating insect hemocyte behavior. Testing of this hypothesis is currently underway.

The identity of molecules transferred through nonjunctional hemichannels is rarely known (Schalper et al. 2008), although paracrine signaling by hemichannel-mediated transfer of small signaling compounds, such as ions, cyclic nucleotides and ATP, can be important (Stout et al. 2002; Zhao et al. 2005; Locovei et al. 2006). In preliminary examinations of High Five cells, no significant alteration in extracellular ATP levels was observed following LPS application (Luo, preliminary data), despite reduction in dye uptake. However, a more rigorous examination of possible signaling molecules including ATP and other likely candidates (Ca^2+^, Mg^2+^, cyclic nucleotides, lipid derivates, etc.) must be pursued. Additionally, large molecules may be capable of traversing insect gap junctions (Cieniewicz and Woodruff. 2008), suggesting that previously unconsidered molecules may also utilize hemichannels.

In conclusion, we have demonstrated for the first time the activity and subsequent modification of nonjunctional hemichannels in insect cells. Given the widespread use of insect cells for recombinant protein expression, and the functional analogy between both hemichannels and gap junctions comprised by innexins, pannexins, and connexins, these data support the use of insect cells in future studies of gap junction biology. Identification of the specific components transferred by hemichannels, though difficult, should be a key goal. In addition, characterizing the role of hemichannels in specific physiological systems of insects, particularly during ontogenic processes and immune responses, should also inform fundamental research into modes of cellular communication in those systems.

## Abbreviations

**CBX**,: carbenoxelone;
**DAPI**,: 4′-6-Diamidino-2-phenylindole;
**DCC**,: divalent cation containing;
**DCF**,: divalent cation free;
**EB**,: ethidium bromide;
**FFA**,: flufenamic acid;
**HBSS**,: Hank's buffered salt solution;
**LPS**,: lipopolysaccharide;
**LY**,: lucifer yellow;
**MFO**,: mefloquine;
**PI**,: propidium iodide;
**RD**,: rhodamine dextran;
**SFM**,: serum free media
